# Evaluating the Cardiometabolic Efficacy and Safety of Lipoprotein Lipase Pathway Targets in Combination with Approved Lipid-Lowering Targets: A Drug Target Mendelian Randomization Study

**DOI:** 10.1161/CIRCGEN.124.004933

**Published:** 2025-03-07

**Authors:** Eloi Gagnon, Dipender Gill, Dominique Chabot, Héléne T. Cronjé, Shuai Yuan, Stephen Brennan, Sébastien Thériault, Stephen Burgess, Benoit J. Arsenault, Marie-Joe Dib

**Affiliations:** 1 Centre de recherche de l’Institut universitaire de cardiologie et de pneumologie de Québec, Université Laval, Québec (QC), Canada; 2 Sequoia Genetics LTD., Translation & Innovation Hub, 84 Wood Lane, London, England; 3 Department of Medicine, Faculty of Medicine, Université Laval, Québec (QC), Canada; 4 Department of Public Health, Section of Epidemiology, University of Copenhagen, Denmark; 5 Unit of Cardiovascular and Nutritional Epidemiology, Institute of Environmental Medicine, Karolinska Institutet, Stockholm, Sweden; 6 Department of Neurology, Mater Misericordiae University Hospital, Dublin, Ireland; 7 Department of Molecular Biology, Medical Biochemistry and Pathology, Faculty of Medicine, Université Laval, Québec (QC), Canada; 8 MRC Biostatistics Unit, University of Cambridge, Cambridge, UK; 9 Cardiovascular Epidemiology Unit, Department of Public Health and Primary Care, University of Cambridge, Cambridge, UK; 10 Cardiovascular Division, Perelman School for Advanced Medicine, University of Pennsylvania, Philadelphia, USA

**Keywords:** drug target MR, Mendelian randomization, lipoprotein lipase pathway

## Abstract

**Background:**

Therapies targeting the lipoprotein lipase (LPL) pathway are under development for cardiometabolic disease. Insights into the efficacy − both alone and in combination with existing lipid-lowering therapies − modes of action, and safety of these agents are essential to inform clinical development. Using Mendelian randomization (MR), we aimed to (i) evaluate efficacy (ii) explore shared mechanisms (iii) assess additive effects with approved lipid-lowering drugs, and (iv) identify secondary indications and potential adverse effects.

**Methods:**

We selected triglyceride lowering genetic variants located in the genes encoding ANGPTL3, ANGPTL4, APOC3 and LPL and conducted drug target MR on primary outcomes including coronary artery disease (CAD) and type 2 diabetes (T2D), and secondary outcomes including apolipoprotein-B (apoB), waist-to-hip ratio (WHR), body mass index, and 233 metabolic biomarkers. We conducted interaction MR analyses in 488,139 UK Biobank participants to test the effect of combination therapy targeting the LPL and low-density lipoprotein receptor pathways. Finally, we investigated potential secondary indications and adverse effects by leveraging genetic association data on 1,204 disease endpoints.

**Results:**

Genetically predicted triglyceride lowering through the perturbation of LPL pathway activation targets ANGPTL4, APOC3, and LPL was associated with lower risk of CAD and T2D, and lower apoB. Genetically predicted TG-lowering through ANGPLT4 was associated with lower WHR, suggestive of a favourable body fat distribution. There was no evidence of multiplicative interaction between genetically proxied perturbation of ANGPTL4, APOC3, and LPL and that of HMGCR and PCSK9 on CAD and T2D, consistent with additive effects. Lastly, associations of genetically predicted LPL pathway targeting were supportive of the broad safety of these targets.

**Conclusions:**

Our findings provide genetic evidence supporting the efficacy and safety of LPL pathway activation therapies for the prevention of CAD and T2D, alone or in combination with statins or PCSK9 inhibitors.

## Abbreviations

APOB: Apolipoprotein B
APOC3: Apolipoprotein
CIII ANGPTL4: Angiopoietin-like 4
BMI: Body mass index
CAD: Coronary artery disease
GRS: Genetic risk score
HMGCR: HMG-CoA reductase
LDL-c: Low-density lipoprotein cholesterol
LPL: Lipoprotein lipase
LDLR: Low density lipoprotein receptor
MR: Mendelian randomization
PCSK9: Proprotein convertase subtilisin/kexin type 9
T2D: Type 2 diabetes
TG: Triglyceride
WHR: Waist-to-hip ratio

## Introduction

Cardiovascular disease (CVD) is the leading cause of morbidity and mortality worldwide. CVD prevention strategies include the management of hypercholesterolemia, predominantly through low-density lipoprotein cholesterol (LDL-c) lowering medications including statins and proprotein convertase subtilisin/kexin type 9 (PCSK9) inhibitors. Alternative lipid-lowering therapies targeting the lipoprotein lipase (LPL) pathway are emerging as efficacious in the management of hypertriglyceridemia^1–3^. A recent phase 3 clinical trial demonstrated that plozasiran, an RNA interference agent targeting apolipoprotein C3 (APOC3), a known inhibitor of LPL, significantly reduced triglyceride levels in patients with mixed hyperlipidemia^4^. Similarly, findings from a phase 2b clinical trial indicated that zodasiran, an RNA interference agent targeting angiopoietin-like 3 (ANGPTL3), another LPL inhibitor, also led to a significant reduction in triglyceride levels in patients with mixed hyperlipidemia^5^. Nonetheless, pharmacological activators of LPL are still in the development stages for the prevention of CVD, and our understanding of their efficacy in treating cardiometabolic disease alone or in combination with existing lipid-lowering therapies remains incomplete.

LPL is an enzyme that catalyzes the clearance of atherogenic triglyceride (TG)-rich lipoprotein particles. Currently, genetic evidence supports a potentially causal relationship between enhanced LPL-mediated lipolysis and a reduced risk of coronary artery disease (CAD) and type 2 diabetes (T2D)^6–8^. Specifically, gain-of-function genetic variants in the *LPL* gene region, and loss-of-function (LoF) variants in the genes encoding its known inhibitors angiopoietin-like 4 *(ANGPTL4)* and apolipoprotein CIII *(APOC3)* are associated with a lower risk of coronary artery disease (CAD)^9–14^. More recent studies have also suggested an association between *ANGPTL4* LoF variants and a lower risk of T2D^9,15^, in addition to a more favourable body-weight distribution^9^. In contrast to LDLR-pathway drugs, genetically predicted LPL pathway targeting scaled on apoB levels have heterogeneous associations with CAD, suggesting that the association of the LPL pathway with CAD is not entirely explained by apoB lowering^6^. There is therefore a need to investigate the mechanisms underlying the heterogeneity in biomarkers and potential differential effects.

Large outcome trials are needed to confirm the cardio-protective effects of targeting the LPL pathway. In light of this, we can harness human genetics to streamline the identification of indications and adverse effects and prioritize relevant causal biomarkers that could inform clinical development efforts. Genetic variants predicting the perturbation of pharmacological targets can be used in the drug target Mendelian randomization (MR) paradigm to rapidly and cost-effectively investigate on-target drug effects^16,17^. The random allocation of genetic variants at conception means that this approach is less vulnerable to bias from environmental confounding and reverse causation that can hinder causal inference in traditional epidemiological studies. Although MR studies have investigated the effects of LPL pathway activation on CAD and T2D liability, they have been limited to specific outcomes, and their modes of action, drug target interactions, and safety have not been comprehensively investigated.

The overarching aim of this study is to provide translationally relevant evidence with implications for ongoing clinical development of pharmacological interventions targeting the LPL pathway. We thereby employ a comprehensive MR framework to investigate LPL pathway targets for their efficacy in reducing CAD and T2D liability, both independently and in combination with existing approved lipid-lowering targets. We further assess similarity in their modes of action and interrogate potential novel indications and adverse effects using genetic association data for biomarkers and clinical outcomes.

## Methods

The UK Biobank is a large-scale prospective study gathering deep-phenotype records and genetic data of >500 000 individuals aged between 40 and 69 years at baseline and recruited between 2006 and 2010 in the United Kingdom. UK Biobank received approval from the British National Health Service, North West - Haydock Research Ethics Committee (16/NW/0274). All participants of UK Biobank provided informed consent at the baseline assessment. Data access permission for this study was granted under UK Biobank application 25205. Access to UK Biobank data can be granted via the Access Management System of the UK Biobank (https://www.ukbiobank.ac.uk/enable-your-research/apply-for-access). This study was approved by the Institutional Review Board of Ethic Commity of the Quebec Heart and Lung Institute under protocol number 2019-3124,21673, ensuring compliance with ethical guidelines and regulations._The code used to perform this analysis is available at https://github.com/gagelo01/LPL_pathway. *Following the transparency and openness promotion guidelines*, GWAS summary statistics were all retrieved in the public domain in publication listed in the Supplemental Table 1.

### Study Design

**Figure 1:**
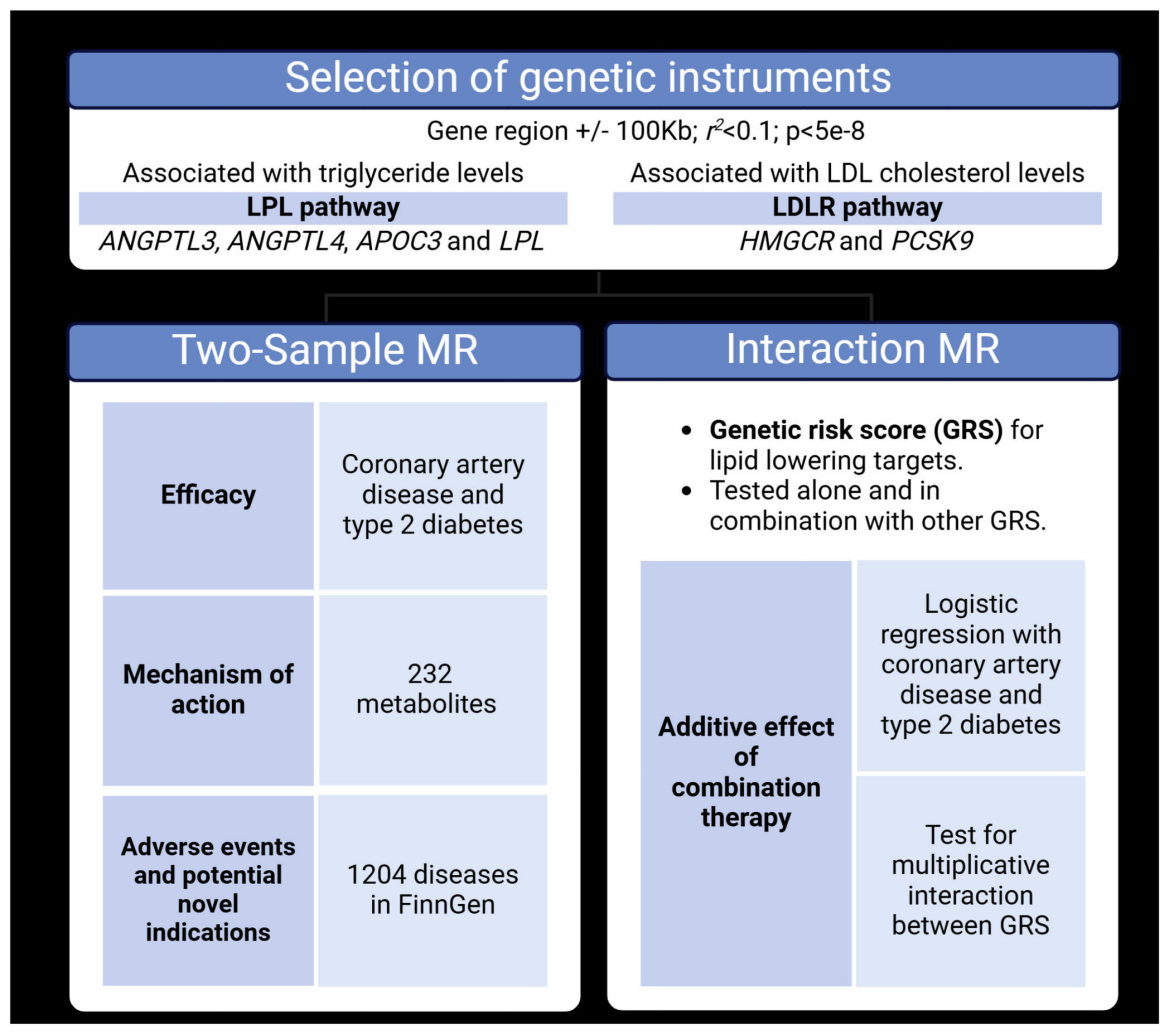
Schematic overview of the study design. ANGPTL3 = Angiopoietin-like 3; ANGPTL4 = Angiopoietin-like 4; APOC3 = Apolipoprotein CIII; LPL = Lipoprotein lipase; HMGCR = HMG-CoA reductase; PCSK9 = Proprotein convertase subtilisin/kexin type 9; LDLR = Low-density lipoprotein receptor. Created with BioRender.com.

Using MR, we aimed to (i) investigate the efficacy of LPL pathway drug targets on cardiometabolic outcomes; (ii) explore similarities in mechanisms of action across these targets; (iii) assess for additive effects with approved therapies for lipid-lowering targets; and (iv) investigate potential secondary indications and adverse effects. An overview of the study methods is presented in Figure 1.

We first explored the association of genetically proxied TG-lowering through the perturbation of LPL pathway drug targets with CAD and T2D as primary outcomes. We extended our outcomes of interest to apoB, body mass index (BMI) and waist-hip ratio (WHR) in secondary analyses. We then conducted a two-sample MR investigation testing the association with 232 circulating metabolites to gain mechanistic insight into the similarity of the modes of action underlying the identified associations. We then investigated whether targeting the LPL pathway can offer additional cardiovascular benefits in combination with existing lipid-lowering therapies using an MR interaction analysis framework in the UK Biobank. Lastly, we further explored novel indications and adverse effects through a two-sample MR investigation of 1,204 disease endpoints.

### Genetic instruments selection

Our exposures included TG-lowering through LPL pathway drug targets (ANGPTL3, ANGPTL4, APOC3, and LPL), and LDL-c lowering through LDLR pathway drug targets (HMGCR, PCSK9). TG levels were used to select instruments for LPL pathway targets since the main function of LPL is to hydrolyze TG in lipoproteins ^18^, and LDL-c levels were used for LDLR targets since the main function of LDLR is to mediate the endocytosis of cholesterol-rich LDL particles^19^.

We used the GWAS of TG and LDL-c levels measured in 1,494,170 participants of mixed genetic ancestry from the Global Lipids Genetics Consortium (GLGC)^20^. Log-transformed measures of TG and measures of LDL-c were adjusted for age, sex, principal components of population stratification and study-specific covariates. The resulting residuals were normalized using an inverse-rank normal transformation prior to GWAS.

Genetic variants at the following gene regions (GRCh37/hg19) were considered: *ANGPTL4* (chr19:8,429,039-8,439,254), *APOC3* (chr11:116,700,623-116,703,788), *LPL* (chr8:19,796,764-19,824,770), *PCSK9* (chr1:55,505,221-55,530,525), *HMGCR* (chr5:74,632,993-74,657,941). We selected common (MAF>0.1) genetic instruments that were 100 Kb upstream or downstream of their respective gene region, that associated with TG-for ANGPTL3,ANGPLT4, APOC3 and LPL- or LDL-c -for PCSK9 and HMGCR- at genome-wide significance level (P<5e-8), and that were clumped using a pair-wise linkage disequilibrium (LD) cut-off of *r^2^* <0.1. The 1000 genome reference panel of European ancestry was used to perform clumping and finding proxies^21^. Selected genetic instruments, and their association estimates for their corresponding exposures are presented in Supplementary Table 2. We used the F statistic to quantify instrument strength ^22^, and the R^2^ value to quantify variance explained by the genetic instruments^23^.

### Data sources for outcome variables in drug target MR analyses

All GWAS used in this study were publicly available and were selected based on the sample size and the ratio of cases and controls for greatest expected statistical power. GWAS sources are summarized in Supplementary Table 1.

#### Primary and secondary outcomes

As primary outcomes, we selected CAD and T2D. For CAD, we used the GWAS meta-analysis from the CARDIoGRAMplusC4D and UKB (N_cases_=181,522 and N_controls_=1,165,690)^24^. For T2D, we used GWAS meta-analysis of DIAGRAM and UKB (N_cases_=242,283 and N_controls_=1,569,730)^25^.

Secondary outcomes included BMI, WHR and apoB levels, and were selected on the basis of a priori genetic evidence supporting associations between each of these traits with the LPL pathway^9^. We used GWAS summary statistics from a meta-analysis of the Genetic Investigation of Anthropometric Traits (GIANT) consortium and the UK Biobank European ancestry for BMI (N=806,834) and WHR (N=697,734)^26^. These anthropometric measurements were self-reported, measured in a laboratory or measured in a healthcare setting. Measures were corrected for age, age squared, sex, principal components, and study sites. The resulting residuals were transformed to approximate normality with SD of 1 using inverse-ranked normal transformation. For apoB levels, we used GWAS summary statistics from the UK Biobank of 439,214 individuals of European ancestry^27^. Measures were adjusted for age, sex, genotyping chip and the resulting residuals were normalised using inverse rank normal transformation prior to GWAS. The GWAS was performed using a linear mixed model (LMM) algorithm using the BOLT-LMM software to account for population stratification and cryptic relatedness.

#### Phenome-wide outcomes

For other diseases, we included all diseases in FinnGen data freeze 10 with over 1,000 cases totalling 1,204 distinct diseases. FinnGen is a population-based cohort totalling 412,181 genotyped individuals of European ancestry ^28^. Cases were established with electronic health record ICD10 codes. Given the relatively high median age of participants (63 years) and the substantial fraction of hospital-based recruitment, FinnGen is enriched for disease endpoints. All endpoints were adjusted for sex, age, genotyping batch and ten first principal genetic components as covariates prior to GWAS. All GWAS were performed using the SAIGE (v.0.35.8.8) algorithm^29^.

#### Metabolome-wide outcomes

To assess mechanisms of action, we included 232 metabolites quantified nuclear magnetic resonance spectroscopy in up to 136,016 participants from 33 cohorts ^30^. Measures were adjusted for age, sex, study-specific covariates and the resulting residuals were normalised using inverse rank normal transformation prior to GWAS.

### Data sources for interaction drug target MR analyses

The UK Biobank is an ongoing prospective study that includes over half a million volunteers recruited from the general population of the United Kingdom between March 2006 and August 2010. Baseline data includes lipids and lipoprotein levels as well as anthropometric measurements. The date of diagnosis of diseases is available through Hospital Episode Statistic with ongoing follow up. This database uses diagnostic codes from the International Classification of Diseases (ICD)-9^th^ and 10th revision and surgical procedure codes of OPSC (Office of Population, Censuses and Surveys Classification of Interventions and Procedures, versions 3 and 4). Data was downloaded on 2023-02-01. Access to the UK Biobank was granted under application 25205.

#### Outcome definition

We defined CAD, and T2D using Hospital Episode Statistics ICD10 and OPCS4 codes as well as primary and secondary cause of death in death registries. CAD cases were defined as participants with ICD-10 codes for MI (I21.X, I22.X, I23.X, I24.1, or I25.2), other acute ischemic heart diseases (I24.0, I24.8-9) or chronic ischemic heart disease (I25.0-25.1, I25.5-25.9), OPSC-4 codes for coronary artery bypass grafting (K40.1- 40.4, K41.1-41.4, K45.1-45.5), for coronary angioplasty, with or without stenting (K49.1- 49.2, K49.8-49.9, K50.2, K75.1-75.4, K75.8-75.9). T2D cases were defined as participants with non-insulin-dependent diabetes mellitus (E11, E12, E13, E14). We included as cases every participant with the disease before or after the recruitment, as genetic risk scores represent a lifelong risk factor. There were 24,785 CAD cases and 40,930 T2D cases.

### Two-sample MR analysis

As the primary method for MR analysis, we performed the inverse-variance weighted (IVW) method with multiplicative random effects ^31^. We also performed four different robust MR methods : the MR Egger ^32^, the contamination mixture ^33^, the weighted median, and the MR-PRESSO^34^. These methods make different assumptions about the underlying nature of pleiotropy and can be used in combination to assess robustness to pleiotropy ^35^. The association was deemed to be statistically robust to pleiotropy when estimates were directionally consistent and nominally significant (P <0.05) across all methods. We also used other statistics to evaluate compliance to assumptions. To assess heterogeneity, we used the Cochran’s Q. Correction for multiple testing was performed using the false discovery rate (FDR) Benjamini-Hochberg method. We report regression estimates as β and 95% confidence intervals (CI) or odds ratios (OR) and 95%CI for the putative effects of TG and LDL-c lowering on continuous and categorical outcomes, respectively. Estimates represent the changes in outcomes per standard deviation (SD) change in genetically predicted plasma logTG or LDL-c levels.

### Bayesian colocalization analyses

We evaluated the posterior probability that both the lipid-lowering target and the outcome shared a single variant, using a Bayesian model implemented in *coloc* R package ^36^. We included all SNPs 100Kb downstream and upstream of the gene region. We used the default priors for the analysis. We used a posterior probability H4 > 0.80 as a threshold to suggest that the two associations shared the same causal variant within the cis region.

### Drug target interaction MR analyses

#### Instrumental variables

Genetic data was available for 488,139 participants (94% European ancestry) and >28 million genetic markers directly genotyped or imputed using the Haplotype Reference Consortium and UK10K. For drug target interaction MR analyses, each participant was assigned a predicted score of their drug target activity using a genetic risk score (GRS). Five GRS were calculated, for *ANGPTL4, APOC3, LPL, HMGCR*, and *PCSK9*. The GRS were calculated by using the same variants as for the Two Sample MR analyses. A GRS was calculated for each participant by summing the number of risk alleles across all selected SNPs weighted on the strength of their association with either TG for LPL pathway targets or LDL-c for *HMGCR* and *PCSK9*. All GRS were subsequently standardized to have a mean of zero and a standard deviation of one.

#### Statistical analyses

To evaluate the potential synergistic effects of targeting the LPL pathway in combination with existing targets of the LDLR pathway, we conducted a series of analyses using the GRS computed for each drug target in the UK Biobank. Associations of each genetically proxied lipid-lowering target with CAD and T2D were first computed separately, then in combination with all other GRS using interaction terms.

We assessed associations of genetically predicted lipid lowering through each drug target with risk of CAD and T2D using Cox regression adjusted for sex, and the first 10 principal components of ancestry. Association with LDL-c and TG were investigated using linear regression models adjuted for age at recruitment, sex and the first 10 principal components of ancestry. Follow-up was started at birth. To investigate synergistic effects on CAD and T2D, each LPL pathway GRS (ANGPLT4, APOC3, and LPL) was tested in combination with the LDLR pathway GRS (HMGCR and PCSK9) using interaction terms, assessing their independent (additive) or synergistic (multiplicative) effects on each outcome. A 5% false discovery rate (FDR) threshold was used to account for multiple comparisons.

## Results

### Genetically predicted association of LPL pathway drug target perturbation on primary and secondary outcomes

Figure 1 presents an overview of the study methods. In two-sample drug target MR analyses, activation of the LPL pathway was mimicked through ANGPTL3, ANGPTL4, APOC3, and LPL perturbation using 20, 9, 30 and 35 genetic instruments explaining 0.3%, 0.3%, 1.7% and 1.4% of the variation in TG levels, respectively. For drug targets activating the LDLR pathway, we leveraged 24 variants that accounted for 0.3% of the variation in LDL-c levels for HMGCR, and 41 variants that account for 0.9% of the variation in LDL-c levels for PCSK9. The genetic instruments used to mimic perturbation of lipid-lowering targets all had an F statistic above 10.

**Figure 2:**
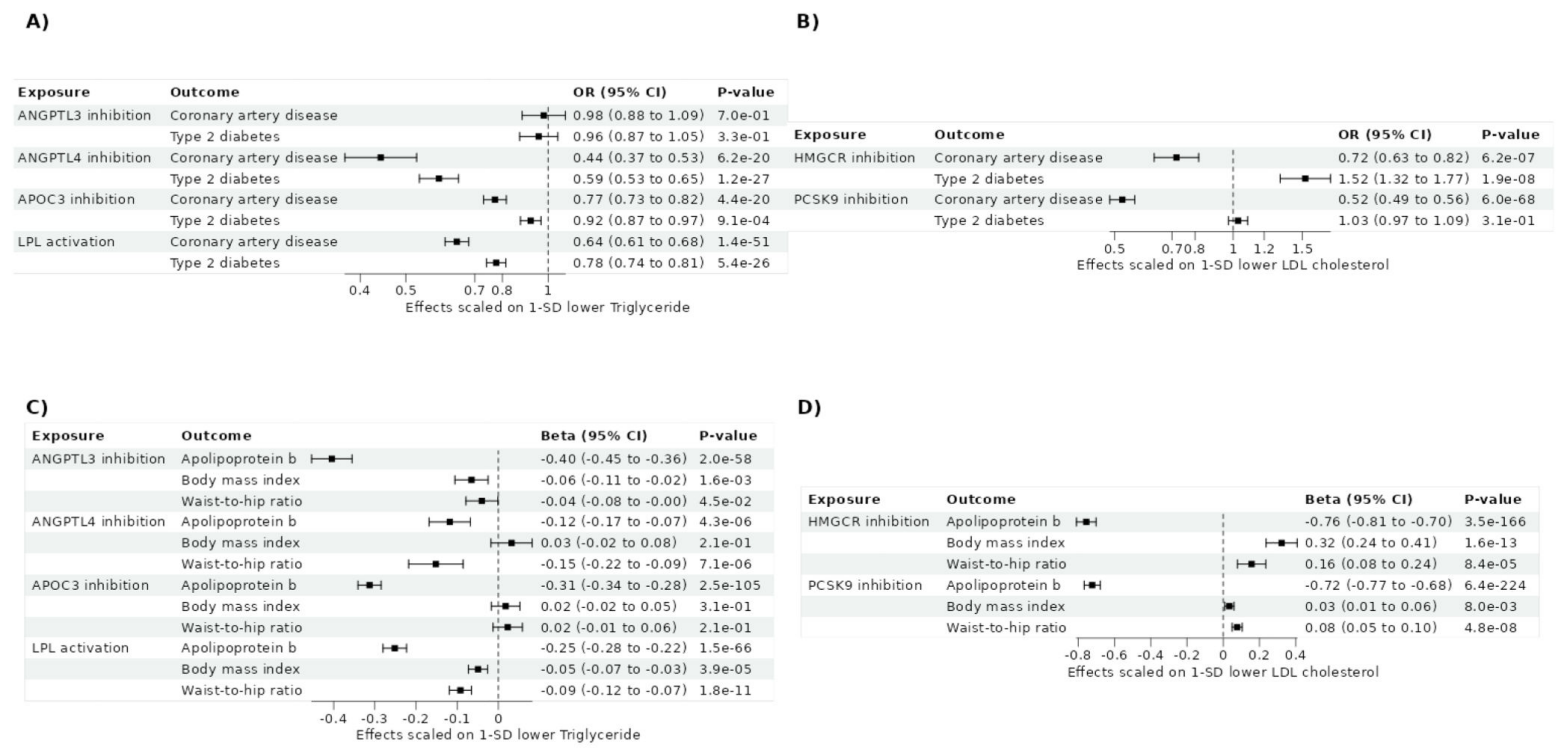
Association between genetically predicted lipid-lowering targeting and cardiometabolic traits and diseases using Two sample Mendelian randomization. A) Impact of LPL pathway activation on coronary artery disease and type 2 diabetes. B) Impact of PCSK9 and HMGCR inhibition on coronary artery disease and Type 2 diabetes. C) Impact of LPL pathway activation on ApoB and anthropometric traits. D) Impact of PCSK9 and HMGCR inhibition on ApoB and anthropometric traits. ANGPTL3 = Angiopoietin-like 3; ANGPTL4 = Angiopoietin-like 4; APOC3 = Apolipoprotein CIII; LPL = Lipoprotein lipase; HMGCR = HMG-CoA reductase; PCSK9 = Proprotein convertase subtilisin/kexin type 9.

Considering primary outcomes, genetically predicted perturbation of the LPL pathway through APOC3, ANGPTL4, and LPL was associated with lower risk of CAD and T2D (Figure 2A; Supplemental Tables 3-4) after adjusting for multiple testing. Genetic colocalization supported that CAD shared a causal variant with ANGPTL4 and APOC3 (PPH4>0.99), but not with LPL (PPH4=0.003). The posterior probability that LPL and CAD had distinct causal variant was 0.99. By contrast, ANGPTL3 inhibition was not associated with CAD or T2D. Genetically predicted inhibition of *PCSK9* was associated with lower risk of CAD but not T2D, and genetically predicted inhibition of HMGCR was associated with lower risk of CAD, but higher risk of T2D (Figure 2B).

Considering secondary outcomes, our results indicated that targeting of both the LPL and LDLR pathways were associated with lower apoB levels (Figure 2C-D). Genetically predicted TG-lowering through *ANGPLT4* was associated with lower WHR, but not BMI, whereas genetically predicted TG-lowering through *LPL* was associated with lower BMI and WHR. There were no significant associations for genetically proxied APOC3 perturbation and BMI and WHR. (Figure 2C). These results were supported by MR methods more robust to the inclusion of pleiotropic variants (Supplemental Table 4). Genetic colocalization supported that ApoB shared a causal variant with ANGPTL3, ANGPTL4 and APOC3 (PPH4>0.99), but not with LPL (PPH4<4e-10) (Supplemental Table 5).

### Metabolome-wide associations of genetically proxied LPL and LDLR pathway perturbation

To inform on the similarity of mode of action, we assessed the associations of genetically predicted lipid-lowering drug targeting and 233 metabolites measured with high-throughput nuclear magnetic resonance (Supplemental Tables 6-7).

**Figure 3:**
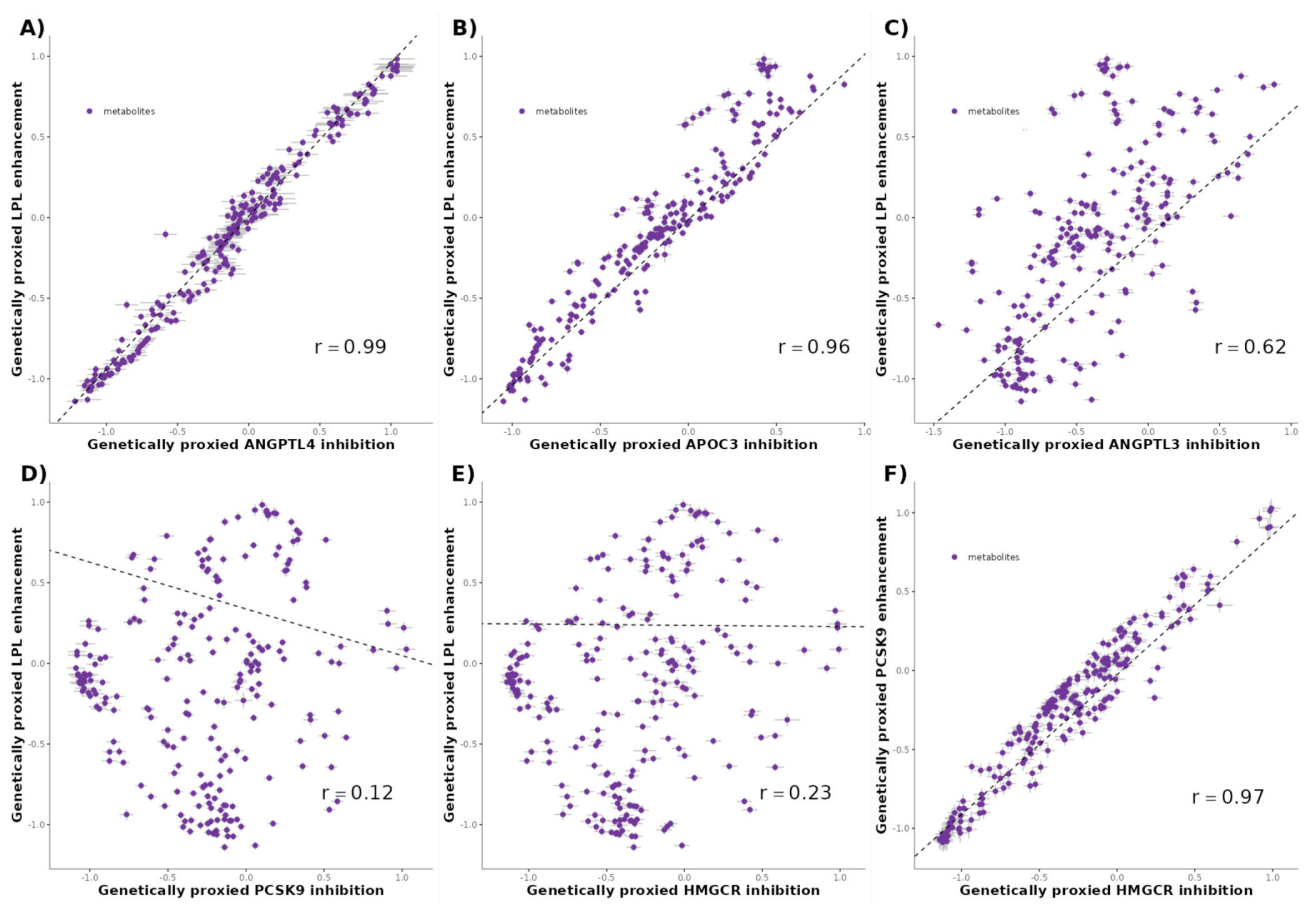
Association between genetic enhancement of LPL and metabolites in relation with the association of genetic inhibition of ANGPTL4 and APOC3 on the same metabolites. Each effect is scaled to a one standard deviation effect on TG levels. Panel A) ANGPTL4 inhibition vs. LPL enhancement. Panel B) APOC3 inhibition vs. LPL enhancement. Panel C) ANGPTL3 inhibition vs. LPL enhancement. Panel D) PCSK9 inhibition vs. LPL enhancement. Panel E) HMGCR inhibition vs. LPL enhancement. Panel F) PCSK9 inhibition vs. HMGCR inhibition. The dashed line represents the best fitting line scaled on the inverse of the variance of the estimates ANGPTL3 = Angiopoietin-like 3; ANGPTL4 = Angiopoietin-like 4; APOC3 = Apolipoprotein CIII; LPL = Lipoprotein lipase; HMGCR = HMG-CoA reductase; PCSK9 = Proprotein convertase subtilisin/kexin type 9.

Genetically predicted ANGPTL3, ANGPTL4, APOC3 and LPL perturbation were associated with 207, 169, 216, and 213 distinct metabolites, respectively. LPL pathway targets displayed very similar association with metabolites, with genetically predicted perturbation of LPL and ANGPTL4 exhibiting high levels of correlation between associated metabolites (Pearson’s correlation = 0.99) (Figure 3A; Supplemental Table 8). The same was true for APOC3 and LPL (r = 0.96)(Figure 3B) as well as ANGPTL4 and APOC3 (r = 0.93). ANGPTL3 and LPL had moderately similar association with metabolites (r = 0.62) (Figure 3C). Genetically predicted perturbation of LDLR pathway targets HMGCR and PCSK9 also exhibited a similar association with metabolites (Pearson’s correlation = 0.97) (Figure 3F). Conversely, LPL pathway targets and LDLR-pathway targets had distinct association with metabolites (r ≤0.26) (Figure 3D-E).

### LDLR and LPL pathway drug target interaction

To test the putative effect of combination therapy targeting the LDLR and LPL pathways respectively, we conducted drug target interaction MR analyses using individual-level data in UK Biobank.

We first validated the GRS computed using selected genetic instruments for each drug target. We found that the GRS for ANGPTL3, ANGPTL4, APOC3, and LPL were robustly associated with TG levels in the UK Biobank (P<1.6e-164) (Supplemental Table 9). The GRS for HMGCR and PCSK9 were also robustly associated with LDL-cholesterol levels (P<1.1e-161) (Supplemental Table 9).

**Figure 4:**
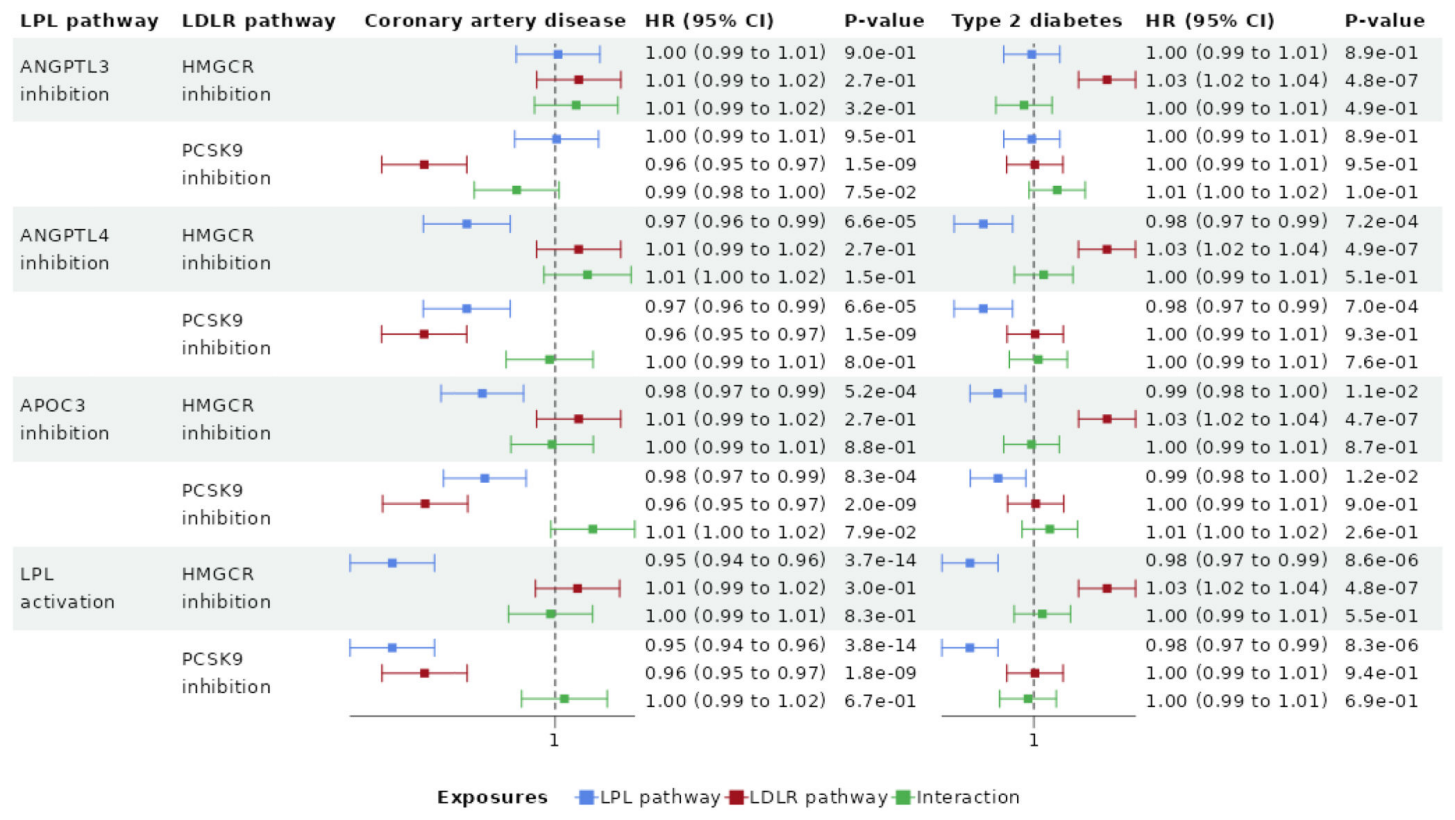
Interaction between pair of genetic risk score of LPL pathway targets and LDLR pathway targets. Display of the Mendelian randomization estimates in the genetic interaction analysis between LPL pathway targets and LDLR targets. Each Cox model was adjusted for sex and the ten first principal component of ancestry. ANGPTL3 = Angiopoietin-like 3; ANGPTL4 = Angiopoietin-like 4; APOC3 = Apolipoprotein CIII; LPL = Lipoprotein lipase; HMGCR = HMG-CoA reductase; PCSK9 = Proprotein convertase subtilisin/kexin type 9; LDLR = Low-density lipoprotein receptor.

Figure 4. shows the association of LPL pathway target GRS, LDLR pathway GRS, and their interaction with the primary outcomes. There was no evidence of multiplicative interaction between each pair of GRS and the incidence of CAD or T2D, consistent with an additive model (Figure 4A, Supplemental Table 10). The associations of ANGPTL4, APOC3, and LPL GRS with CAD and T2D remained consistent in direction and significance when tested in combination with either the HMGCR and PCSK9 GRS, suggesting that LPL pathway activation may be an effective therapeutic target in lowering CAD risk in combination with statin or PCSK9 inhibitor treatment.

### Assessment of secondary indications and adverse effects

**Figure 5:**
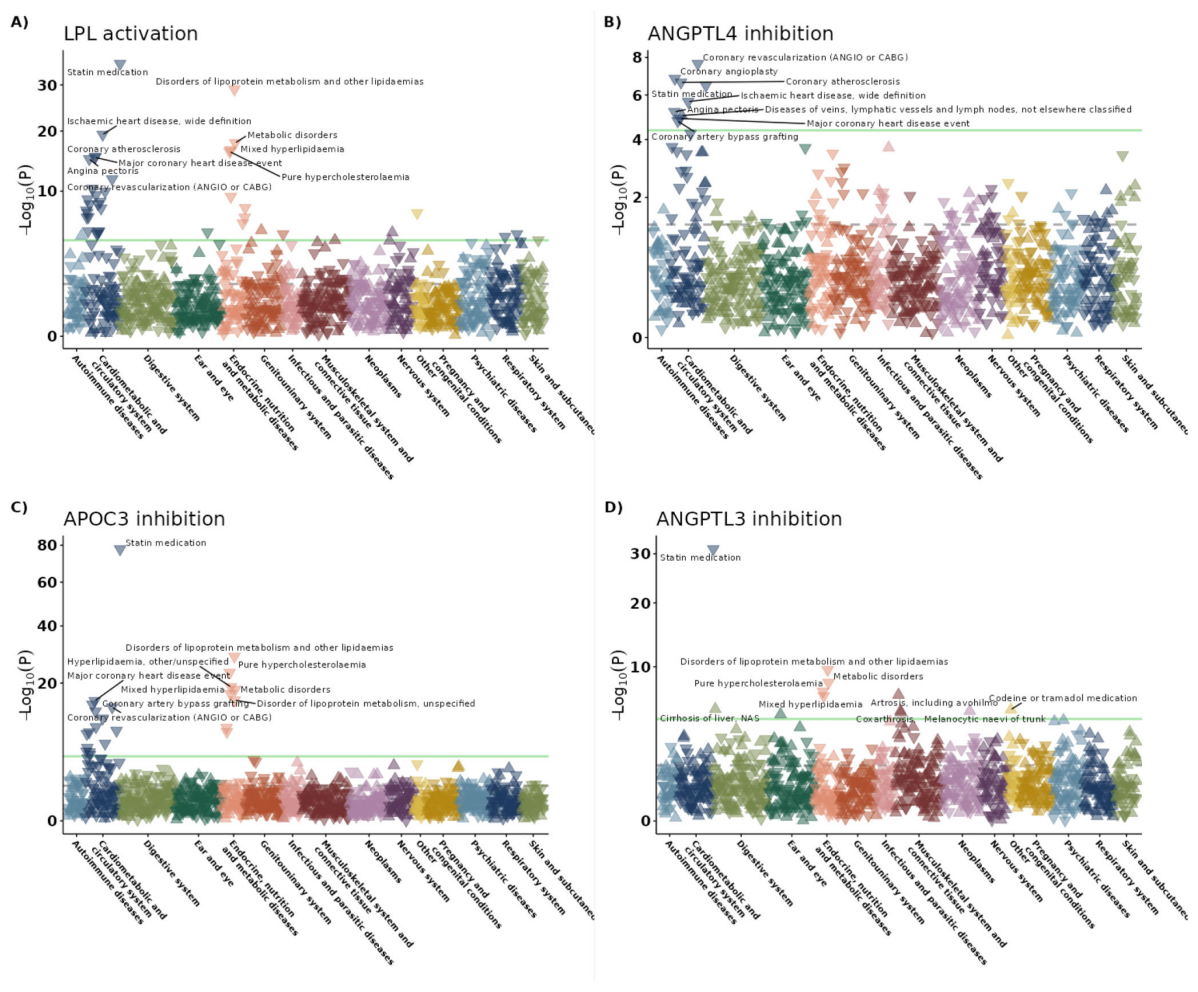
Association between genetically predicted targeting of lipid-lowering targets and 1204 diseases in FinnGen. A) LPL activation, B) ANGPTL4 inhibition. C) APOC3 inhibition. D) ANGPTL3 inhibition. The green line is the threshold for multiple testing significance. Triangles pointing down represent negative associations, whereas triangles pointing up represent positive associations. The ten most significant associations that pass multiple testing correction are annotated. ANGPTL3 = Angiopoietin-like 3; ANGPTL4 = Angiopoietin-like 4; APOC3 = Apolipoprotein CIII; LPL = Lipoprotein lipase.

To assess on-target safety and potential novel indications of LPL pathway activation, we assessed associations with 1,204 clinical outcomes in FinnGen (Figure 5; Supplemental Tables 11-12; Supplemental Figure 1supp). Genetically predicted targeting of ANGPTL4, APOC3 and LPL were associated with a lower risk of CAD, replicating our main MR findings. Genetically predicted LPL activation was associated with lower risk of T2D (OR = 0.80, 95% CI=0.73 to 0.87, P=2.0e-07), but this was not found for APOC3 inhibition (OR = 0.92, 95% CI=0.85 to 1.00, p=0.058). There was suggestive evidence for a beneficial effect of ANGPLT4 inhibition and risk of T2D (OR= 0.70, 95% CI=0.58 to 0.85, P=3.5e-04). Genetically predicted LPL activation was significantly associated with increased risk of Alzheimer’s disease in FinnGen (OR = 1.47, 95% CI=1.24 to 1.74, P=7.6e-06). However, when attempting replication using a better-powered GWAS meta-analysis of Alzheimer’s disease ^37^ the association was null (OR = 1.00, 95% CI=0.98 to 1.03, P=8.4e-01). The strongest unfavourable association of genetically predicted ANGPTL3 inhibition was with arthrosis (OR = 1.37, 95% CI=1.23 to 1.53, p=1.1e-08). The strongest unfavourable association of genetically predicted ANGPTL4 inhibition was “diseases of veins, lymphatic vessels and lymph nodes, not elsewhere classified” (OR = 1.41, 95% CI=1.21 to 1.64, P=9.4e-06). There was suggestive evidence for an association between genetically predicted *APOC3* inhibition and idiopathic pulmonary fibrosis (OR = 1.56, 95% CI=1.17 to 2.08, P=2.5e-03), although not passing the multiple testing threshold.

## Discussion

To our knowledge, this is the first MR study to comprehensively investigate perturbation of the LPL pathway by simultaneously exploring similarities in modes of action, potential synergistic effects with existing approved lipid-lowering drug targets, and secondary indications. We showed that genetically predicted activation of the LPL pathway through APOC3, ANGPTL4, or LPL was associated with lower risk of CAD and T2D, likely through similar pathways. We also provide data to support the efficacy of targeting the LPL pathway in treating CAD and T2D, either as stand-alone or in combination with licensed lipid-lowering drug targets, with evidence of additive, but not multiplicative effects. Additionally, our findings support the broad safety of LPL pathway targeting. Altogether, these results provide genetic evidence that the LPL pathway may represent safe and efficacious targets to prevent cardiometabolic diseases, as a stand-alone or combination therapy.

### Efficacy of LPL pathway drug targets in treating CAD and T2D

Our findings highlight associations of genetically predicted TG-lowering in the LPL pathway with lower CAD and T2D risk, corroborating findings from previous clinical trials. For instance, the antisense oligonucleotide against APOC3 olezarsen is associated with lower levels of TG and to a lesser extent apoB in participants with normal^1^ or elevated levels of TG^2^ and at high cardiovascular risk^3^, in line with our Mendelian randomization results. Genetic colocalization showed that LPL and ApoB’ probability of sharing a causal variant was only 4e-10. Genetic colocalization makes the assumption that there is only one causal variant in the gene region. The low PPH4 may be explained by the existence of many causal variants in the gene region. Similarly, our findings showed that HMGCR targeting was associated with a higher BMI and risk of T2D. Concordantly, statin medication has been implicated in increased risk of developing T2D^38^ and weight gain^39^. Inhibition of PCSK9 was associated with reduced CAD risk but showed no significant associations with T2D risk, aligning with previous clinical findings on lipid-lowering therapies. These concordant results provide external validation for our genetic instrument selection strategy to proxy long-term perturbations of lipid-lowering targets.

### Similarity in metabolic modes of action among LPL pathway drug targets

Our results show that when scaled on TG levels, the association between genetically predicted ANGPTL4 and CAD risk was greater in magnitude than for that of LPL activation and CAD risk, suggesting that the effect of ANGPTL4 on CAD may only be partly independent of LPL activation. Since ANGPTL4 is an inhibitor of LPL^40^, it has been proposed that any effect of ANGPTL4 inhibition on CAD would be mediated by LPL activation ^41^. In support of this notion, a genetic analysis showed that the E40K as a proxy of ANGPTL4 inhibition, and rs115849089 as a proxy of LPL activation have very similar effects on metabolites (r>0.98) ^41^. Using our instrument selection strategy and other metabolite datasets, we obtained a broadly similar correlation coefficient (r>0.99), strongly suggesting that ANGPTL4 potentially impacts these plasma metabolites exclusively via LPL. If LPL may entirely mediate the effect of ANGPTL4 on metabolites, that may not be the case for CAD. In our study, the association between genetically predicted inhibition of ANGPTL4 and CAD (OR = 0.44, 95% CI=0.37 to 0.53, p=6.2e-20) was significantly higher than that of LPL (OR = 0.64, 95% CI=0.61 to 0.68, p=1.4e-51), suggesting that ANGPTL4 may not only affect CAD through LPL, but also through independent pathways. These pathways could include insulin sensitivity^42^, healthier body fat distribution^43^, and tissue-specific activation of ANGPTL4 in the heart^44^.

ANGPTL3 was not associated with CAD despite being an inhibitor of LPL. Our results show that ANGPTL3 and LPL had quite distinct associations with metabolites, suggesting that ANGPTL3 may have functions beyond inhibition of LPL. The most notable differences were related to HDL metabolism. For example, while LPL enhancement was positively associated with APOA1 levels (0.65 95% CI= 0.58 to 0.71, p=4.1e-96), ANGPTL3 inhibition association was in the opposite direction (-0.65 95% CI=-0.73 to -0.57, p=8.4e-57). Concordantly, Inhibition of ANGPTL3 in humans decrease HDL cholesterol levels ^5^. This dual role may explain why genetically predicted ANGPTL3 inhibition is not associated with CAD. Another explanation may be that statistical power was too low to detect an association of genetically predicted ANGPTL3 inhibition on CAD risk.

### Genetic evidence supporting additive rather than interactive effects of LPL and LDLR pathway drugs

Our results show no robust evidence of multiplicative interaction between each pair of LPL pathway drug target GRS and LDLR GRS for incidence of CAD and T2D. These data should not be interpreted as definitive evidence that combination therapy will result in additive effects. Indeed, the GRS explained only a small proportion of the variance of lipids, limiting power to detect interaction despite the use of continuous (instead of dichotomized) GRSs, aiming to maximise power^45^. Well-powered RCT will be needed to test whether drugs targeting the LPL pathway could yield additional cardiovascular protection when given in combination with established lipid lowering targets. Nonetheless, our interaction MR data as well as recent data showing that these targets may have an effect on CAD that is independent of apoB ^6^ support this notion.

### Assessment of safety and secondary indications

In our study, the strongest unfavourable association of genetically predicted ANGPTL4 inhibition was “diseases of veins, lymphatic vessels and lymph nodes, not elsewhere classified”. Concordantly, antibodies against ANGPTL4 result in lymphadenopathies in mice and monkey ^11^. Similarly, *ANGPTL4* knock out mice develop a lethal phenotype that includes lymphadenopathy ^46^. However, the development of lymphadenopathies are not observed in mice with *ANGPTL4* liver specific knockout ^47^ or adipose tissue-specific knockout^48^, suggesting that tissue-specific targeting of ANGPTL4 may not display this side effect. The safety of tissue-specific ANGPTL4 inhibition is further supported by a recent randomized, double-blind, placebo-controlled Phase I study evaluating the safety of a GalNAc conjugated ASO against ANGPTL4, with no reported lymphadenopathy ^49^.

In our results, there was a strong and significant association with LPL activation and an increased risk of Alzheimer in FinnGen that was not replicated in a large GWAS meta-analysis. The reason for this discrepancy could be because the association in Finngen is biased away from the null due to the exclusion of other dementia cases, possibly introducing collider bias. Alternatively, the association in the GWAS meta-analysis could be biased towards the null, due to the inclusion of Alzheimer’s by proxy cases (i.e., participants with a biological parent with Alzheimer’s disease included as cases), leading to potential biased estimates. Contrasting our results, other studies have shown a potential protective effect of LPL activation on Alzheimer’s disease. For example, mutations decreasing LPL function have been associated with increased Alzheimer’s disease risk^50^. Similarly, microglia-specific LPL knockout mice have exacerbated cognitive deficits compared to control mice^51^. Full elucidation of the role of LPL in Alzheimer’s disease will require further research beyond these genetic analyses.

### Strengths and Limitations

To our knowledge, this is the largest study to investigate the putative causal effects of LPL pathway drug targets on cardiometabolic outcomes, and the first study to provide insights into potential modes of action, interactive effects with existing lipid-lowering drugs, and potential secondary indications and adverse effects using an MR framework. Notably, our MR study design relies on multiple robust and independent cis-acting instruments, thereby minimizing the likelihood of confounding and horizontal pleiotropy. However, as with all studies, our findings should be interpreted within the context of their limitations. Firstly, our genetic data predominantly included individuals of European ancestry, thereby limiting the generalizability of our findings to other populations. Second, the genetic risk scores used in the interaction MR analyses explained a low proportion of variance of lipid levels, limiting our ability to detect interactions. Additionally, several study samples used to derive our exposure and outcome genetic associations included the same cohorts, leading to sample overlap which could potentially increase the risk of type I errors^52^. It has been shown that sample overlap will bias estimates towards the observational association estimates in a relation proportional to the amount of sample overlap and inversely proportional to the strength of the instruments^52^. The mean F statistics for each target were high (>10), with mean F statistics ranging from 196 to 899 across exposures, mitigating bias from sample overlap^52^. Lastly, MR estimates are suggestive of potential effects of genetic predisposition of an exposure on an outcome of interest but do not provide definitive evidence that any given pharmacological intervention targeting the exposure will change the outcome. Similarly, MR instruments are used as a proxy of lifelong genetic effects, which may differ from pharmacologic interventions that may occur over shorter time periods with different potency.

## Conclusions

In conclusion, these results support that LPL pathway targeting may reduce the risk of CAD, and in contrast to other established lipid-lowering drug targets, may also reduce the risk of T2D. Further, genetically predicted LPL pathway targeting generally appeared safe with evidence consistent with additive effects on top of statin and PCKS9 inhibiting therapies. These insights from human genetic data may be used to inform and prioritise clinical development efforts.

## Supplementary Material

Supplementary material

## Data Availability

All data used in this study are in the public domain. Supplementary Table 1 describes the data used and relevant information to retrieve the summary statistics. Code to reproduce the results of this manuscript is available on https://github.com/gagelo01/LPL_pathway
