# Supplementary material for: Evaluating the Cardiometabolic Efficacy and Safety of Lipoprotein Lipase Pathway Targets in Combination with Approved Lipid-Lowering Targets: A Drug Target Mendelian Randomization Study
Source: Circ Genom Precis Med. Author manuscript; Available in PMC 2025 Apr 9. (PMC7617573; doi:10.1161/CIRCGEN.124.004933)
Supplement: Supplementary material [file EMS204261-supplement-Supplementary_material.pdf]

Supplemental Figures

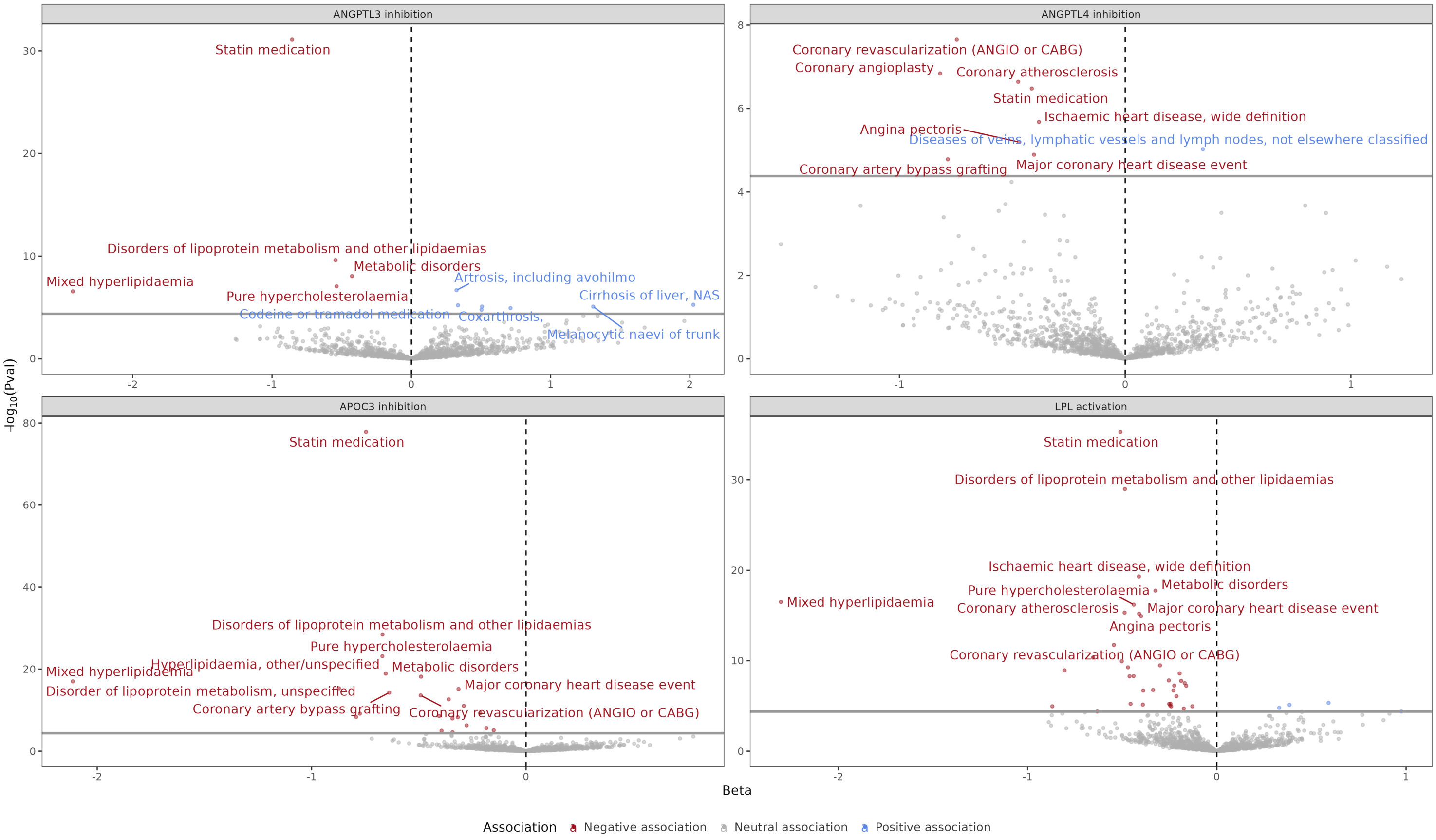

Supplemental Figure 1. Association between genetically predicted targeting of lipid-lowering targets and 1204 diseases in FinnGen. A) ANGPTL4 gene region in 100KB window. B) APOC3 gene region in 100KB window. C) LPL gene region in 100KB window. D) PCSK9 gene region in 100KB window. The ten most significant associations that pass multiple testing correction are annotated. ANGPTL3 = Angiopoietin-like 3; ANGPTL4 = Angiopoietin-like 4; APOC3 = Apolipoprotein CIII; LPL = Lipoprotein lipase.
